# Assessing the Use of Media Reporting Recommendations by the World Health Organization in Suicide News Published in the Most Influential Media Sources in China, 2003–2015

**DOI:** 10.3390/ijerph15030451

**Published:** 2018-03-05

**Authors:** Xin Chu, Xingyi Zhang, Peixia Cheng, David C. Schwebel, Guoqing Hu

**Affiliations:** 1 Department of Epidemiology and Health Statistics, Xiangya School of Public Health, Central South University, Changsha 410078, China; chuxin0027@sina.com (X.C.); zhangxingyi@csu.edu.cn (X.Z.); chengpeixia92@163.com (P.C.); 2 Department of Psychology, University of Alabama at Birmingham, Birmingham, AL 35294, USA; schwebel@uab.edu

**Keywords:** suicide, suicide prevention, media reporting

## Abstract

Public media reports about suicide are likely to influence the population’s suicidal attempts and completed suicides. Irresponsible reports might trigger copycat suicidal behaviors, while responsible reports may help reduce suicide rates. The World Health Organization (WHO) released recommendations to encourage responsible suicide reports in 2008. However, little is known about whether these recommendations are reflected in the suicide news for most countries, including China. In this study, we assessed the responsibility of suicide stories published in the most influential newspaper and Internet media sources in China from 2003 to 2015, using the media reporting recommendations by the World Health Organization (WHO). In total, 3965 and 1836 eligible stories from newspaper and Internet-based media, respectively, were included in the study. Newspapers and Internet-based media performed similarly in applying WHO recommendations to report suicide news. Three recommendations were applied in over 88% of suicide stories. However, four recommendations were seldom applied, including offering information about where to seek help and linking the suicide event to mental disorders. Government and the journalism industry should work together to improve media reporting of news about suicide in China.

## 1. Introduction

Suicide is a major public health issue. According to World Health Organization statistics, suicide claims approximately one million lives globally each year, and represents the second leading cause of death in 15–29-year-olds [1]. Among all potential contributing factors, media reporting has repeatedly been shown to impact individual suicidal behavior [2]. In particular, suicide stories in the mass media are associated with clusters of suicides through a “contagion” or “Werther” effect, especially among young people [3]. In these cases, irresponsible suicide reports in the media trigger copycat behavior among vulnerable readers [4]. In contrast, responsible media reports could serve as educational material for the public, supporting those at risk and helping to reduce suicide rates [4].

Several developed countries have developed national strategies to improve alignment of news media items with the best practice standards for reporting. For example, New Zealand convened a round table comprising the media and researchers to formulate press release guidelines [5]. Similarly, in partnership with media and mental health organizations, Australia provides technical support for media organizations [6]. Based on previous work, the World Health Organization (WHO) and the International Association for Suicide Prevention (IASP) released *Preventing Suicide: A Resource for Media Professionals* in 2008 and promoted it as a guide for proper reporting of suicide news internationally [7]. Some studies have assessed the impact of implementing either national or WHO guidelines on suicide rates. Sonneck et al. [8], for example, reported that subway suicide rates in Vienna decreased by 75% from 1980 to 1990 following the implementation of national guidelines in Austria. Similarly, Niederkrotenthale et al. [9] examined subway suicide rates from 1982–2005 in Austria and found that the number of suicides reduced by 81 annually after the media guideline was introduced. Three other studies report that the quality of media reports on suicide improved after the introduction of national media guidelines [6,10,11]. Despite these published findings, other surveys indicate that many journalists are unfamiliar with recommended guidelines in the UK [12] and New Zealand [13,14]. Few published studies examine suicide reporting in the media of low- or middle-income countries.

With 21% of the world’s population, China is estimated to account for 16% of global suicides in 2016 [15]. However, little is known about how suicide events are reported in the Chinese media. Fu and Yip [16] assessed 2279 reports from 15 newspapers in Hong Kong, Taiwan, and Guangzhou (China) over 6 months in 2010 and found that WHO recommendations were seldom applied in suicide articles. Feng et al. [17] examined the compliance of 2015 newspaper reports about suicide with WHO media guidelines in Guangdong province and found the majority of suicide reports did not comply. Chiang et al. [18] investigated front-page reporting about suicide in four major newspapers in Taiwan between 2001 and 2012 and concluded that reporting of suicide news by the four major Taiwanese newspapers could be improved through regular assessment and observance of the WHO recommendations. These previous studies are limited by focusing on particular regions in China, many of which are higher-income regions outside the mainland People’s Republic of China. Nationwide data have not been analyzed, nor have data across many years.

We conducted this study to examine in greater detail the extent to which suicide news reported by the 10 most influential domestic newspaper and Internet-based public media sources in China reflect WHO recommendations. We considered publications appearing between 2003 and 2015.

## 2. Materials and Methods

### 2.1. Ethical Statement

The study was designed as a retrospective review. All data were acquired from the public domain and the research involved screening only of publicly available newspaper reports and articles. The research was considered exempt from inclusion of human participants and approved by the Ethics committee of Xiangya School of Public Health, Central South University, China (approval number: 201610533051).

### 2.2. Search Strategies

Adopting the approach from Li et al. [19], we developed 20 search terms, including one core word (suicide), four synonyms, and 15 hyponyms of suicide and entered them into search archives for four major Chinese domestic newspaper databases (Wise News (http://wisenews.wisers.net), Apabi (http://www.apabi.com/), CNKI (http://www.cnki.net/), and People’s Daily Online (http://www.People.com.cn/)), plus the domestic Internet-based media database Baidu News (http://news.baidu.com).

Considering that newspapers with low circulation often directly reprint news from those with higher circulation (i.e., the top national newspapers) [20], we selected the 10 most influential newspapers based on circulation for inclusion. All have a circulation >1.20 million per issue [21]. Four circulate nationally and six regionally. These newspapers were Global Times, Reference News, People’s Daily, Qilu Evening News, Yangtse Evening Post, Xinhua Daily Telegraph, Qianjiang Evening News, Chutian Metropolis Daily, Southern Metropolis Daily, and Peninsula Metropolitan Daily.

The top 10 Internet-based media sources were determined according to the Alexa Ranking, an online tool ranking websites based on multiple usage indicators. The 10 Internet-based media were Gmw.cn (http://www.gmw.cn/), CCTV.com (http://www.cctv.com/), Youth.cn (http://www.youth.cn/), QQ.com (http://www.qq.com/), Ifeng.com (http://www.ifeng.com/), NetEase (http://www.163.com/), China.com.cn (http://www.china.com.cn/), Sina (http://www.sina.com.cn/), Xinhuanet (http://www.xinhuanet.com/), and People.cn (http://www.people.com.cn/).

### 2.3. Inclusion Criteria

Considering the availability and accessibility of the included newspapers and Internet-based media resources through electronic datasets or online search engines, we limited the searches to news published from 1 January 2003 to 31 December 2015. Media reports prior to 2003 and after 2015 were excluded for two reasons: (1) online full-text media reports before 2003 in China are not covered by any database, and (2) media reports after 2015 are missing for some newspapers in the electronic datasets we used. Reports meeting the following criteria were included: (1) they were published in Mainland China, (2) they reported completed suicides or suicide attempts, and (3) they were nonfictional. News about the same suicide event reported by two or more media sources were considered independent reports.

### 2.4. Outcome Measure

We used 12 dichotomous items (yes/no) to assess the reporting of included suicide news based on the WHO media reporting recommendations [7], which consist of the following:

a. Acknowledge the link between suicide and mental disorders like depression;
b. Discuss possible impacts on survivors and victim’s families in terms of psychological suffering and stigma;
c. Avoid language that sensationalizes or normalizes suicide;
d. Avoid presenting suicide as a solution to problems;
e. Avoid prominent placement like the front page, boxes or similar;
f. Avoid undue repetition of stories about suicide;
g. Avoid explicit description of the method;
h. Avoid labeling any sites as hot spots for suicide;
i. Word headlines carefully;
j. Avoid photographs or video footage of the deceased, the means or the scenes;
k. Show due consideration for people bereaved by suicide and respect their privacy;
l. Provide information about where to seek help.

### 2.5. Data Extraction

Coding was conducted manually by trained researchers. A sample of 10% was selected randomly for re-assessment by a second independent coder; concordance coefficients for all 12 items were ≥0.91.

### 2.6. Statistical Analysis

Numbers and proportions of suicide stories adhering to the WHO recommendations were calculated. Because the data were population-based, only descriptive statistics were applied. Simple linear regression examined linear trends in proportion of suicide stories adhering to the WHO recommendations over time. *p* < 0.05 was considered statistically significant.

## 3. Results

### 3.1. Screening Eligible Suicide Stories

**Figure 1:**
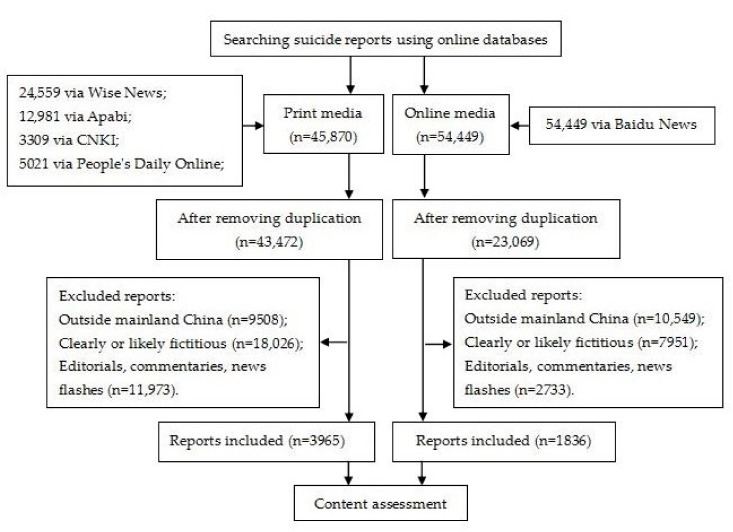
Selection process of media suicide reports.

Over 100,000 records were initially searched (45,870 newspaper reports and 54,449 Internet stories) (Figure 1). After removing duplicate records (2398 for newspaper and 31,380 for Internet) and ineligible stories (39,507 for newspaper and 21,233 for Internet), we conducted a content assessment on 3965 newspaper stories and 1836 Internet-based stories. Stories were ineligible if they described news from outside mainland China; if they were fictitious or deemed likely fictitious by coders; and if they were editorials, commentaries, or news flashes such as one-line headlines or single-sentence “flashes” rather than substantive news stories.

### 3.2. The Proportion of Suicide Stories Adhering to the WHO Recommendations 

**Figure 2:**
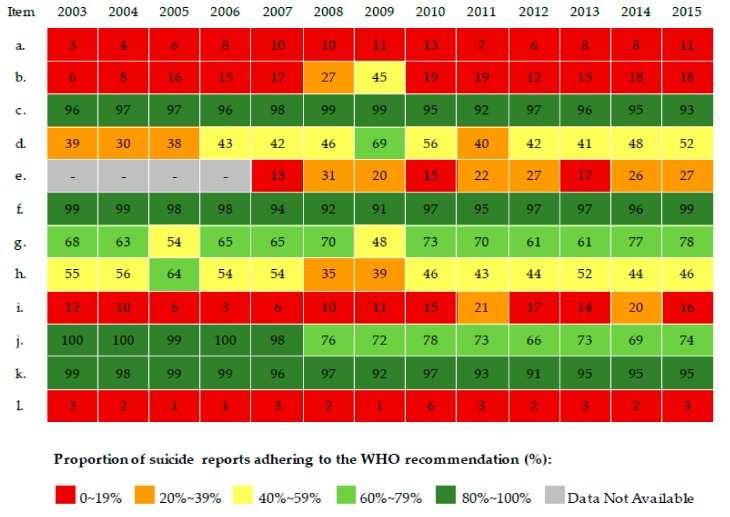
Proportion of suicide news stories applying the WHO recommendations in the top 10 newspapers in China, 2003–2015.

**Figure 3:**
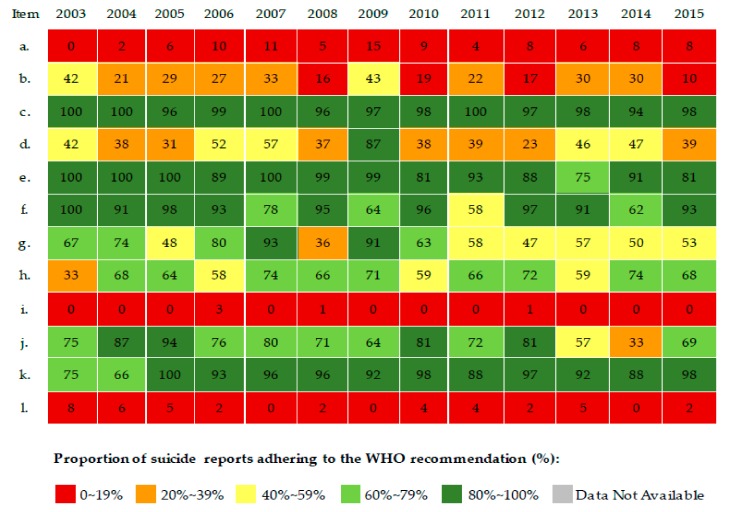
Proportion of suicide news stories applying the WHO recommendations in the top 10 Internet-based media sources in China, 2003–2015.

In general, the proportions (%) of suicide news stories applying each WHO recommendation remained essentially stable from 2003 to 2015, despite occasional outliers from certain years (Table 1, Figure 2 and Figure 3). Because of data unavailability in the databases that we used, data from 2003 to 2006 were absent for item *e,* “*Avoid prominent placement like the front page, boxes or similar*.”

In general, newspapers and Internet-based media performed similarly, with the exception of the WHO recommendation to “avoid prominent placement like the front page, boxes or similar” (item *e* in Table 1)—23% of newspaper stories adhered to this recommendation compared to 86% of Internet-based media stories.

Over 88% of stories followed three recommendations (items *c*, *f*, and *k* in Table 1), in spite of moderately low proportions in items *f* and *k* in certain years for suicide stories by Internet-based media (Figure 3). Four recommendations (items *a*, *b*, *i,* and *l* in Table 1) were seldom applied, with a few minor exceptions (items *b* and *i* were occasionally adhered to in specific years by newspapers; item *b* was occasionally adhered to in specific years by Internet-based media; Figure 2 and Figure 3). 

The remaining four recommendations (*d*, *g*, *h*, and *j* in Table 1) were often applied (a range from 43–84% across newspaper and Internet-based stories). The applied proportions of news stories from both media sources did not show any significant linear trends across the years, *p* > 0.05.

## 4. Discussion

### 4.1. Key Findings

Using freely accessible data, this study assessed adherence to WHO media reporting recommendations for suicide stories published in the most influential newspaper and Internet media sources from 2003 to 2015 in China. We found that the WHO suicide media reporting recommendations were poorly and inconsistently applied by widely distributed newspaper and Internet-based media sources in China. No significant trends emerged to suggest change in reporting practices from 2003 to 2015.

### 4.2. Interpretation of Findings

Our findings generally replicate those of two previous studies analyzing local media reports in China [16,17]. They also generally replicate findings from an analysis in India [22], another middle-income country, although one item—“avoid language which sensationalizes or normalizes suicide”—yielded very different results in our Chinese analysis (96–98% adherence) compared to those in India (31% adherence).

Our findings concerning the degree of compliance with WHO recommendations are very different from results reported from developed countries (note that most research in developed countries is based on national guidelines rather than WHO recommendations, but the national guidelines are extremely similar to WHO guidelines [23,24,25]). For instance, item *a*, “*acknowledge the link between suicide and mental disorders like depression,*” was adhered to in only 8% of suicide stories by Chinese newspapers compared to 12% in US (newspapers) [26], 30.4% in New Zealand (radio, television, newspaper and Internet clippings) [25], and 52.6–76.4% in Australia (newspaper, Internet-based media, TV, and radio) [6]. Similarly, item *g*, “*avoid explicit description of the method*,” was adopted in 62% of Internet-based suicide stories and 65% of newspaper-based suicide stories in China, in comparison to 50.4–86% in Australia, 69% in the United Kingdom (Google News) [27], and 67.6% in New Zealand. Other examples include item *i*, “*word headlines carefully*,” which was adhered to in just 12% of newspaper stories and about 1% of Internet-based stories about suicide in China but in 79.7% of stories in New Zealand and 70.5–78.8% in Australia, and item *l*, “*provide information about where to seek help*,” which ranked last in most studies but was met by just 2–3% of stories in China compared to 6.5–17.7% in Australia and 30.8% in Singapore [28]. Differences might be explained by a variety of factors, including different religious beliefs and cultural practices. Also relevant are national media guidelines and different national patterns of journalist education/training. All Chinese journalists receive similar education, and the central government does not regulate suicide reporting; such patterns differ in most of the countries mentioned above. 

One unanticipated result of our research was the fact that we discovered many stories about fictitious novels that included plots about suicide. Although such stories are not real, they may impact readers and potentially create unwanted suicide risk if they are improperly described. The impact of fictitious novels involving suicide plots on the individual suicide risk of readers merits further research.

### 4.3. Policy Implications

Given the large number of suicide deaths in China (130,556 in 2016 according to Global Burden of Disease 2016 estimates [15]) and research evidence that the introduction of media guidelines can reduce suicide risk by improving media reporting [29], we recommend that the Chinese government consider two strategies to reduce suicide risk to its citizens: (1) translate the WHO’s media reporting recommendations into Chinese and require their use for reporting suicide news in Chinese media sources, and (2) integrate the WHO recommendations into ongoing education and training programs for journalists. 

There may also be value in developing national media guidelines in China that are based on the WHO guidelines. Dialogic communication is believed to be effective to activate both sets of stakeholders, journalists, and policymakers [30]. If government officials could collaborate with media professionals and suicide prevention experts to develop and implement best-practice standards or guidelines for suicide reporting, it may yield stronger results than reliance on WHO guidelines alone. 

### 4.4. Limitations

This study has some limitations. First, the print and online media sources were chosen by circulation and daily page views, and therefore omit media sources with smaller circulations or a small number of views. The exclusion of non-mainstream media likely does not affect our findings significantly, however, because (a) media sources with low circulation often reprint news from high-circulation media sources in China, and (b) all domestic journalists receive highly similar education and training in China, no matter what type of media source they write for. Second, we focused only on traditional print and Internet media sources due to obstacles in collecting complete reports from other sources such as television, radio, or social media. As social media sources such as Facebook, Twitter, blogs, and WeChat grow in popularity and influence among young people, it would be valuable to conduct further studies of social media news reporting to identify opportunities to implement responsible social media reporting practices and ensure access via social media to support those at risk of suicide. Third, we did not interview journalists and editors, individuals who may have provided supplemental insights to our findings. Last, our findings may be affected somewhat by the subjective judgment of raters, although all raters received standard training before they evaluated suicide reports and reliability coefficients among a portion of the sample were strong. 

## 5. Conclusions

From 2003 to 2015, the overall adherence of the Chinese suicide stories with WHO guidelines was mixed. Adherence was adequate on some items but extremely low for other specific recommendations in the WHO guidelines. Media compliance with particular items, such as “acknowledge the link between suicide and mental disorders like depression,” “discuss possible impacts on survivors and victim’s families in terms of psychological suffering and stigma,” “word headlines carefully,” and “provide information about where to seek help” were poor and need to be improved. We noted no significant linear-trends in application of inclusive stories across the 13-year time span we studied. Multiple stakeholders, including the Chinese government, could take action to regulate and enhance suicide reporting in China, to integrate WHO recommendations into national media reporting, and to improve the professional education and training of journalists.

## Figures and Tables

**Table 1:** Proportions of reported suicide news that adhered to WHO recommendations in the 10 most influential newspaper and Internet-based media sources in China, 2003–2015.

WHO Media Report Recommendations	Newspaper (*n* = 3965)			Internet-Based (*n* = 1836)		
	*Pr*	*A*	*B*	*Pr*	*A*	*B*
*a.* Acknowledge the link between suicide and mental disorders like depression	8	3–13	0–18	8	0–15	0–12
*b.* Discuss possible impacts on survivors and victim’s families in terms of psychological suffering and stigma	16	6–45	0–31	25	10–43	20–35
*c.* Avoid language which sensationalizes or normalizes suicide	96	92–99	86–100	98	94–100	97–100
*d.* Avoid presenting suicide as a solution to problems	43	30–69	20–75	45	23–87	20–60
*e.* Avoid prominent placement like the front page, boxes or similar ^†^	23	13–31	17–36	86	75–100	66–100
*f.* Avoid undue repetition of stories about suicide	97	91–99	95–100	89	58–100	70–100
*g.* Avoid explicit description of the method	65	48–78	33–82	62	36–93	30–66
*h.* Avoid labeling any sites as hot spots for suicide	51	35–64	38–91	63	33–74	50–80
*i.* Word headlines carefully	12	3–21	5–32	1	0–3	0–10
*j.* Avoid photographs or video footage of the deceased, the means or the scenes	84	66–100	72–100	69	33–94	58–75
*k.* Show due consideration for people bereaved by suicide and respect their privacy	96	91–99	93–100	93	66–100	80–100
*l.* Provide information about where to seek help	2	1–6	0–47	3	0–8	0–9

*Pr*: Proportion of suicide news stories applying the WHO recommendations. *A*: Minimum and maximum proportion of suicide news applying the WHO recommendation across years. *B*: Minimum and maximum proportion of suicide news applying the WHO recommendation across media sources. ^†^ Newspaper data from 2003 to 2006 were unavailable for item *e*. Note: large ranges for items *b*, *d*, *g*, *h*, *i*, and *k* were due to outliers from certain years or media sources.
